# The presentational flow chart “unwell adult” of the Manchester Triage System—Curse or blessing?

**DOI:** 10.1371/journal.pone.0252730

**Published:** 2021-06-03

**Authors:** Vanessa Brutschin, Monika Kogej, Sylvia Schacher, Moritz Berger, Ingo Gräff

**Affiliations:** 1 Emergency Department, University Hospital Bonn, Bonn, Germany; 2 Emergency Department, Hospital Köln Kalk, Cologne, Germany; 3 Institute for Medical Biometry, Informatics and Epidemiology, University Hospital Bonn, Bonn, Germany; Heidelberg University Hospital, GERMANY

## Abstract

**Background:**

The presentational flow chart “unwell adult” of the Manchester Triage System (MTS) occupies a special role in this triage system, defined as the nonspecific presentation of an emergency patient. Current scientific studies show that a considerable proportion of emergency room patients present with so-called "nonspecific complaints". The aim of the present study is to investigate in detail the initial assessment of emergency patients triaged according to the presentational flow chart "unwell adult".

**Methods:**

Monocentric, retrospective observational study.

**Results:**

Data on 14,636 emergency department visits between March 12^th^ and August 12^th^, 2019 were included. During the observation period, the presentational flow chart "unwell adult" was used 1,143 times and it was the third most frequently used presentational flow chart. Patients triaged with this flow chart often had unspecific complaints upon admission to the emergency department. Patients triaged with the “unwell adult” chart were often classified with a lower triage level. Notably, patients who died in hospital during the observation period frequently received low triage levels. The AUC for the MTS flow chart “unwell adult” and hospitalization in general for older patients (age ≥ 65 years) was 0.639 (95% CI 0.578–0.701), and 0.730 (95% CI 0.714–0.746) in patients triaged with more specific charts. The AUC for the MTS flow chart “unwell adult” and admission to ICU for older patients (age ≥65 years) was 0.631 (95% CI 0.547–0.715) and 0.807 (95% CI 0.790–0.824) for patients triaged with more specific flow charts. Comparison of the predictive ability of the MTS for in-hospital mortality in the group triaged with the presentational flow chart “unwell adult” revealed an AUC of 0.682 (95% CI 0.595–0.769) vs. 0.834 (95% CI 0.799–0.869) in the other presentational flow charts.

**Conclusion:**

The presentational flow chart "unwell adult" is frequently used by triage nurses for initial assessment of patients. Patient characteristics assessed with the presentational flow chart "unwell adult" differ significantly from those assessed with MTS presentational flow charts for more specific symptoms. The quality of the initial assessment in terms of a well-functioning triage priority assessment tool is less accurate than the performance of the MTS described in the literature.

## Introduction

### Background and importance

The Manchester Triage System (MTS) has found widespread use in emergency departments (ED) across Europe, with the broadest application in German EDs [1]. The MTS uses so-called presentational flow charts for urgency classification, which are oriented around the complaints of the emergency patient. Each of these 52 presentational flow charts is based on an algorithm, which assigns the emergency patient to one of five triage levels after answering so-called indicator questions. The assigned level defines the length of time which is tolerable from the patient’s arrival at the ED to the first contact with a doctor. Compared to other triage systems (e.g. the Emergency Severity Index, ESI), the MTS with its 52 presentational flow charts presents a unique approach by not including resource utilization. Principally, the list of presentational flow charts is intended to cover all chief complaints or symptoms. The choice of the appropriate flow chart is determined to a considerable extent by how the patient presents on arrival at the ED.

In the last decade, several studies provided surprising evidence that typical symptoms, such as chest pain in myocardial infarction, do not provide high accuracy or discrimination with respect to diagnosis or prognosis. Dezmann et al. concluded in their review that "atypical" symptoms cannot rule out acute coronary symptoms (ACS), while "typical" symptoms cannot rule it in [2,3].

Two recent studies of patients presenting at the ED with multiple symptoms showed increased resource requirements and hospitalizations, but no evidence of adverse outcomes, such as acute morbidity or mortality [4,5]. The situation is different for emergency patients who present to the ED with atypical or nonspecific symptoms. This group of patients is obviously at high risk for serious consequences and much higher mortality than patients presenting with typical symptoms. Nonspecific complaints were shown to be of prognostic significance, both in terms of diagnostic uncertainty and survival. Therefore, these results indicate that the systematic evaluation of symptoms at triage can provide additional prognostic information [6–8].

In their review, Bingisser and Nickel postulate that, due to the increasingly widespread use of imaging and clinical chemistry, symptom-oriented research has lost ground in many areas of clinical medicine. Since initial assessment at the process apex is still under-researched, symptom-oriented research in emergency medicine obviously has a special significance [9]. In their opinion, the reliance of formal triage tools on "typical case presentation" is of concern. Current scientific studies show that a relevant proportion of emergency room patients present with so-called "nonspecific complaints" [10,11]. These "non-specific complaints", such as fatigue, chills, or loss of appetite, do not receive separate attention in the triage systems.

The "unwell adult" presentation chart occupies a special role in the MTS by defining itself in terms of the nonspecific presentation of the emergency patient [12]. It is one of the most frequently used presentational flow charts and therefore one of the top five flow charts used in the ED [13,14].

The aim of the present study is a detailed investigation of the initial assessment of emergency patients, who were triaged with the presentational flow chart "unwell adult". The study intends to highlight how patient characteristics and clinical parameters behave and which of the patient characteristics and clinical parameters are found in this triage group. Furthermore, the triage using the presentational flow chart "unwell adult" will be evaluated in comparison with the other MTS presentational flow charts. Possible weaknesses regarding validity, strengths or even potential for improvement of the MTS will be identified in order to ultimately ensure the quality of treatment.

## Material and methods

### Setting and study design

This was a single-center retrospective observational study performed at the emergency department (ED) of the University Hospital Bonn, Germany, a tertiary hospital with over 31 departments, 21 institutes and 1,124 beds [15], treating approximately 42,000 emergency patients per year. Gynecologic, obstetric, and pediatric emergencies up to age 16 (with the exception of traumatized children and children with ENT problems) are cared for in other nearby departments.

Triage in the ED at the University Hospital Bonn is a standardized process, which is defined according to quality guidelines. In accordance with the MTS (4^th^ revised extended edition), which is computer-supported and an integral part of the hospital information system (ED Cockpit; Dedalus Health Care Systems Group Bonn, Germany), every emergency patient in the ED is classified into a priority level.

Each patient presenting as an emergency case is first seen by a specially trained nurse and triaged according to the MTS (triage protocol) [1]. All triage nurses were trained in an in-house schooling for MTS prior to working in the ED. The quality of triage is regularly evaluated via audit three times a year. Moreover, the team has an MTS trainer in its ranks who is responsible for the supervision of the MTS triage [1].

The data were collected over five months from March 12^th^ to August 12^th^, 2019. All data were directly related to emergency treatment in the ED and obtained from the triage protocol, e.g. vital signs and Numeric Rating Scale (NRS). Data derived from hospital admission and emergency treatment (death during hospital stay, medical consultation, level of care etc.) were extracted from the patient’s electronic health record.

In this study, we compared various parameters, such as baseline characteristics, MTS urgency levels, waiting time, infectivity, diagnosis according to ICD-10, patient disposition and in-hospital mortality.

### Inclusion and exclusion criteria

Only adult patients (≥18 years old) were included in this study. Furthermore, all presentational flow charts referring to trauma were excluded. In detail, these were the presentational flow charts “assaults”, “burns and scalds” “falls”, “head injury”, “major trauma”, “self-harm” and “torso injury”. Moreover, all non-specific flow charts from the reference group were excluded to allow comparison of the unspecific flow chart “unwell adult” with more specific flow charts. These were “crying baby”, “irritable child”, “unwell child”, “unwell newborn” and “worried parent”.

### Classification of infectious diseases

The included infectious pathogens were 3MRGN, hepatitis A, B and C, VRE, HIV, influenza, MRSA, norovirus, scabies and tuberculosis.

### Statistics and frequency definitions

The continuous variables are presented with their mean values and standard deviations. The different patient groups were compared using t-test. The categorical variables are presented as absolute and relative frequencies. Group comparisons were carried out using Pearson’s Chi-square test.

Prediction of in-hospital mortality, general hospital admission and admission to the intensive care unit (ICU) based on the five MTS levels were assessed by the area under the curve (AUC) of the receiver operating characteristic (ROC) and their 95% confidence interval (CI).

When comparing MTS urgency levels and general hospital admission, we additionally divided the two comparison groups, "unwell adult " and the other presentational flow charts, into two age categories. Thus, we were able to compare young patients with older, more vulnerable patients. An old patient was classified as aged 65 years and older.

All data were evaluated using SPSS (version 26, SPSS Inc., Chicago, IL; USA).

### Ethic statement

The study received approval (No. 252/19) from the chairman of the local ethics committee (Prof. Kurt Racké, Ethics Committee at the Medical Faculty of the University Bonn). Data obtained from the clinical information system may be used in accordance with the code of medical ethics [16] of the General Medical Council. Furthermore, as stipulated by German data protection regulations, the physician may use existing patient data for analyses without explicitly asking for the consent of patient. All collected clinical data evaluated in this study were fully anonymized prior to analysis. The study design is consistent with the Declaration of Helsinki [17].

## Results

### Baseline characteristics

During the observation period, a total of 14,636 patients presented at the ED. The presentational flow chart "unwell adult" was applied in 1,143 cases. Hence, in the 14,636 patients treated in the ED during this period, it was the third most frequently used flow chart with a share of 7.8%. The leading presentational flow chart was “limb problems” (11.5%), followed by "strange behavior" (8.2%).

The mean age of patients triaged with the presentational flow chart "unwell adult" was 54.04 years (y). This population was substantially older than the patients triaged with the other presentational flow charts (mean value 49.96 y). Furthermore, we found statistical differences among these two groups in relation to blood pressure, heart rate, oxygen saturation, temperature, GCS and pain scale [Table 1].

**Table 1:** Baseline characteristics (mean value with standard deviation) of the presentational flow chart “unwell adult” of the MTS in comparison to the other presentational flow charts.

	over-all (n = 14,636)		presentational flow chart “unwell adult”(n = 1,143)		other presentational flow charts (n = 13,493)		p value
**demographics**		n		n		n	
age	50.28 ± 20.64		54.04 ± 19.51		49.96 ± 20.07		< 0.001
gender (male)	50.9%	7,444	52.20%	597	50.7%	6,847	0.549
**clinical parameters**							
respiratory rate (per min)	14.8 ± 2.74	9,127	15.14 ± 3.31	902	14.77 ± 2.67	8,225	0.376
oxygen saturation (%)	97.44 ± 2.38	8,596	97.23 ± 2.5	967	97.47 ± 2.36	7,629	0.003
BP systolic (mmHg)	138.91 ± 22.5	8,215	133.85 ± 24.3	959	139.58 ± 22.17	7,256	< 0.001
BP diastolic (mmHg)	82.35 ± 14.49	8,211	79.07 ± 15.51	959	82.78 ± 14.29	7,252	< 0.001
heart rate (per min)	83.85 ± 17.42	8,611	85.29 ± 17.35	973	83.67 ± 17.42	7,638	0.006
temperature (°C)	36.61 ± 0.73	8,537	36.87 ± 0.96	894	36.57 ± 0.69	7,643	< 0.001
GCS	14.83 ± 1.1	7,757	14.89 ± 0.64	682	14.83 ± 1.1	7,075	0.029
pain (NRS)	2.88 ± 2.11	8,037	1.92 ± 1.98	750	2.98 ± 2.1	7,287	< 0.001

The mean length of stay of patients triaged with the presentational flow chart "unwell adult" in the ED was 213 ± 121 min. In comparison, the mean length of stay of patients triaged with the remaining flow charts was significantly shorter at 169 ± 124 min (p < 0.001).

A noticeable difference between the presentational flow charts was observed in the patients’ disposition. While 48.6% of the patients triaged with the "unwell adult" chart required inpatient treatment, only 30.5% (p < 0.001) of the patients triaged with the remaining charts did.

### MTS urgency levels

**Fig 1:**
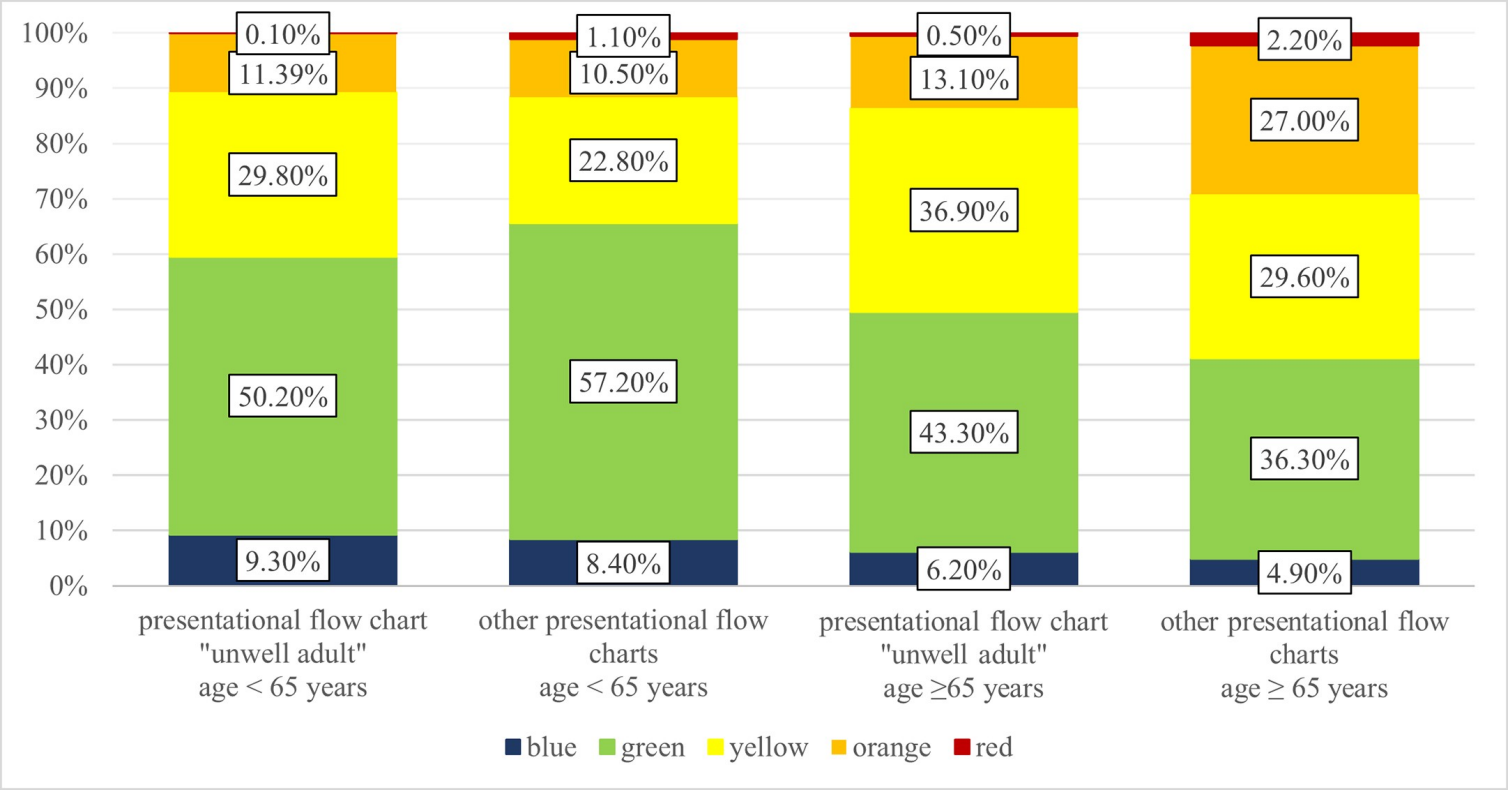
The different MTS urgency levels used in the presentational flow chart “unwell adult” vs. the other presentational flow charts, which were divided into two age categories (aged <65 years and ≥65 years).

The triage revealed a statistically significant difference in the use of priority levels between the two comparison groups “unwell adult” and the other presentational flow charts, which were divided into two age categories (aged <65 years and ≥ 65 years) (p < 0.001) [Fig 1]. Looking at the patients aged ≥ 65 years, it was noticeable that, while the levels "red" and "orange” were used much more frequently in the remaining flow charts (2.20% and 27.00%), 0.5% of patients triaged as “unwell adults” were classified as “red” and 13.10% as “orange”. In this age group, the triage levels "yellow", “green” and "blue" were more frequent at 36.90%, 43.3% and 6.20%, respectively (compared to 29.6%, 36.30% and 4.90% for the other presentational flow charts).

Looking at the patients aged <65 years, the differences in MTS urgency levels did not seem to be quite as pronounced. While patients triaged with “yellow” had a higher proportion in the flow chart “unwell adult” (29.80%) compared to the other flow charts (22.80%), patients triaged with “green” had a lower proportion in the group “unwell adult” than in the comparison group (50.20% vs. 57.29%) (p < 0.001).

### Waiting time and percentage of achieving the predefined MTS time limits

**Fig 2:**
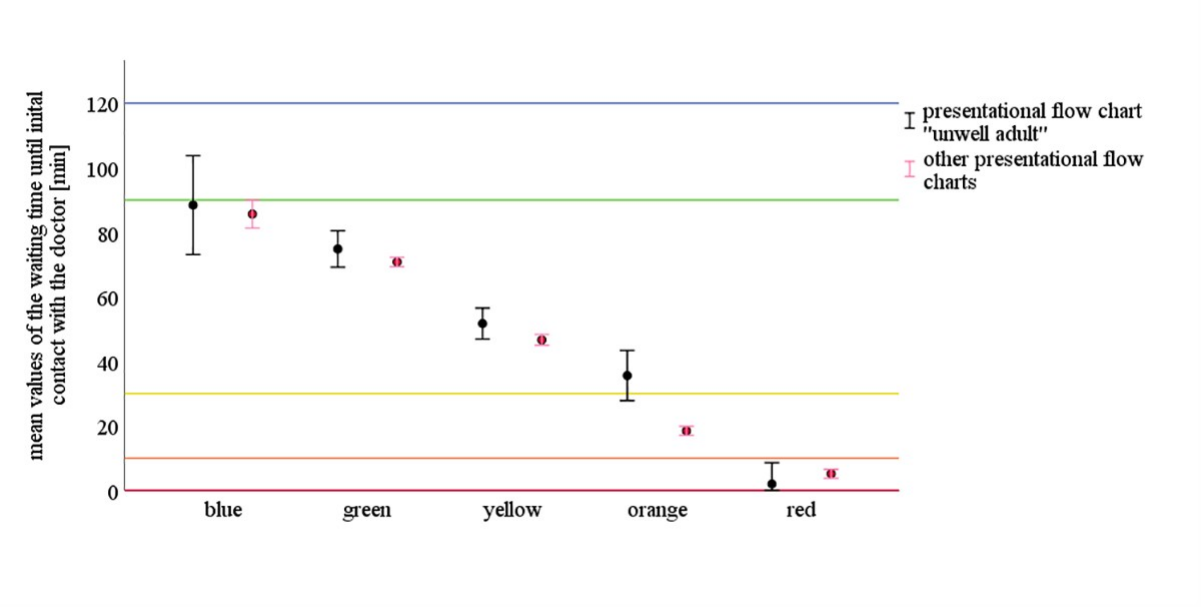
The mean values of the waiting time until initial contact with a doctor with 95% CI for the comparison groups of “unwell adult” and the other presentational flow charts. The colored lines refer to the predefined MTS time limits. Exceedances in the MTS category "red" (immediate contact with a doctor) may be caused by a delayed setting of the "doctor contact timestamp" in the computer system. This is conceivable if, e.g., all personal resources are primarily tied up in direct patient care.

In the MTS triage levels "red", "orange", "yellow", "green" and "blue”, maximum waiting times until the initial contact with a doctor are specified. The evaluation of the percentage degree of achievement (falling below the maximum time) revealed differences between the two groups [Fig 2]. Regarding the lowest MTS levels “blue” and “green”, the mean waiting time in both groups was below the maximum permitted latest contact time with a doctor. In the triage levels "yellow", "orange" and “red”, this was the other way round. Details are shown in Fig 2.

### Infectivity

Of note, patients triaged with the presentational flow chart "unwell adult" were more likely to be infectious at the time of initial hospital admission than patients triaged with the other presentational flow charts (3.7% vs. 1.7%) (p < 0.001).

### Diagnoses according to ICD-10

**Fig 3:**
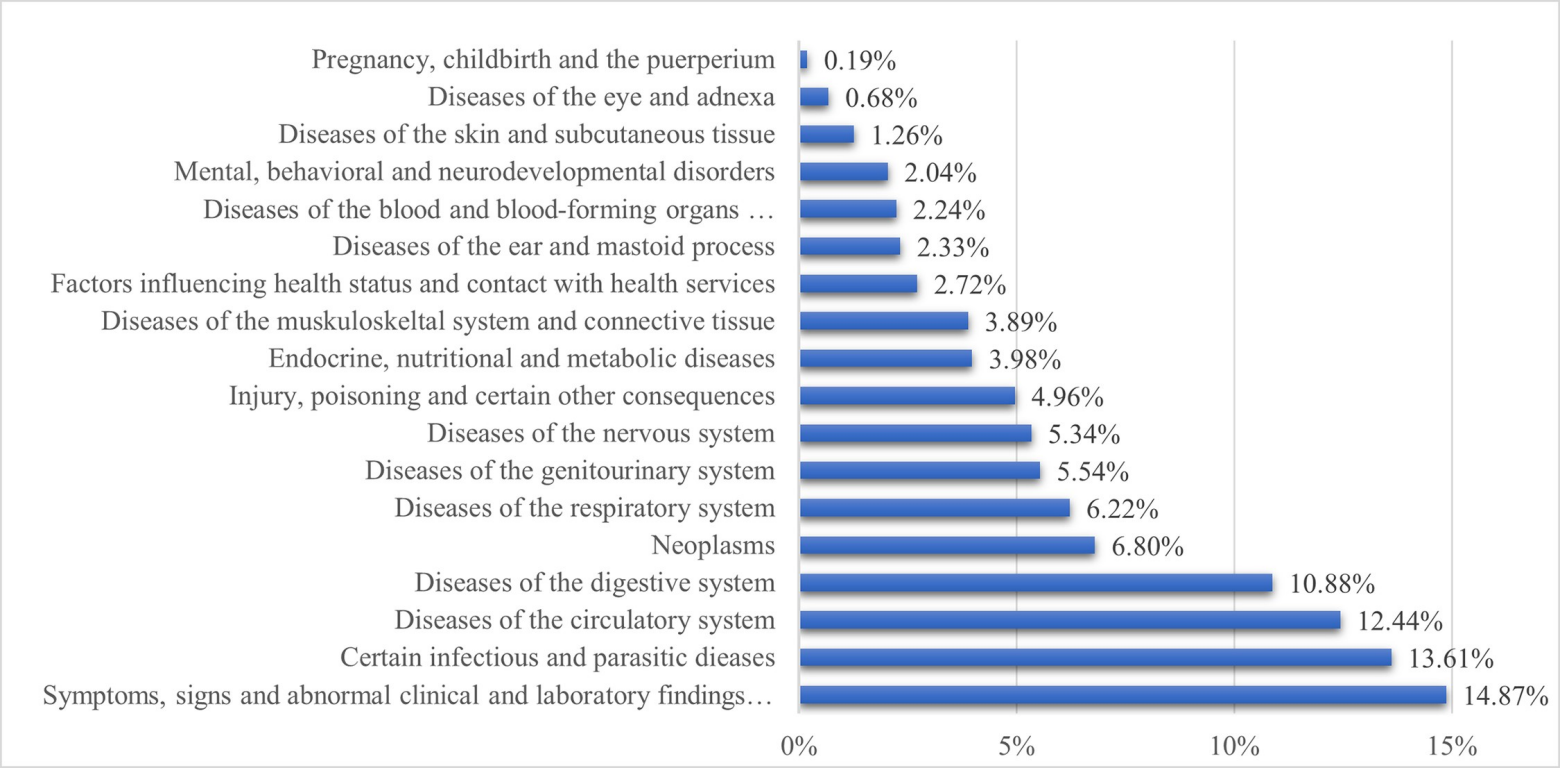
The relative frequencies of the ICD-10 chapters used in the presentational flow chart “unwell adult”.

In patients triaged with the "unwell adult" chart, the leading ICD-10 chapter was "symptoms, signs and abnormal clinical and laboratory findings not classified elsewhere" (14.87%) [Fig 3], followed by the ICD-10 chapters "certain infectious and parasitic diseases" (13.61%), “diseases of the circulatory system” (12.44%), and “diseases of the digestive system” (10.88%). In fifth place, with 6.8%, was "neoplasms". The evaluation of the Canadian Emergency Department Information System (CEDIS) presenting complaint list shows the same pattern. "General and other complaints" were by far the most frequently registered complaint at admission.

### Patient disposition

**Fig 4:**
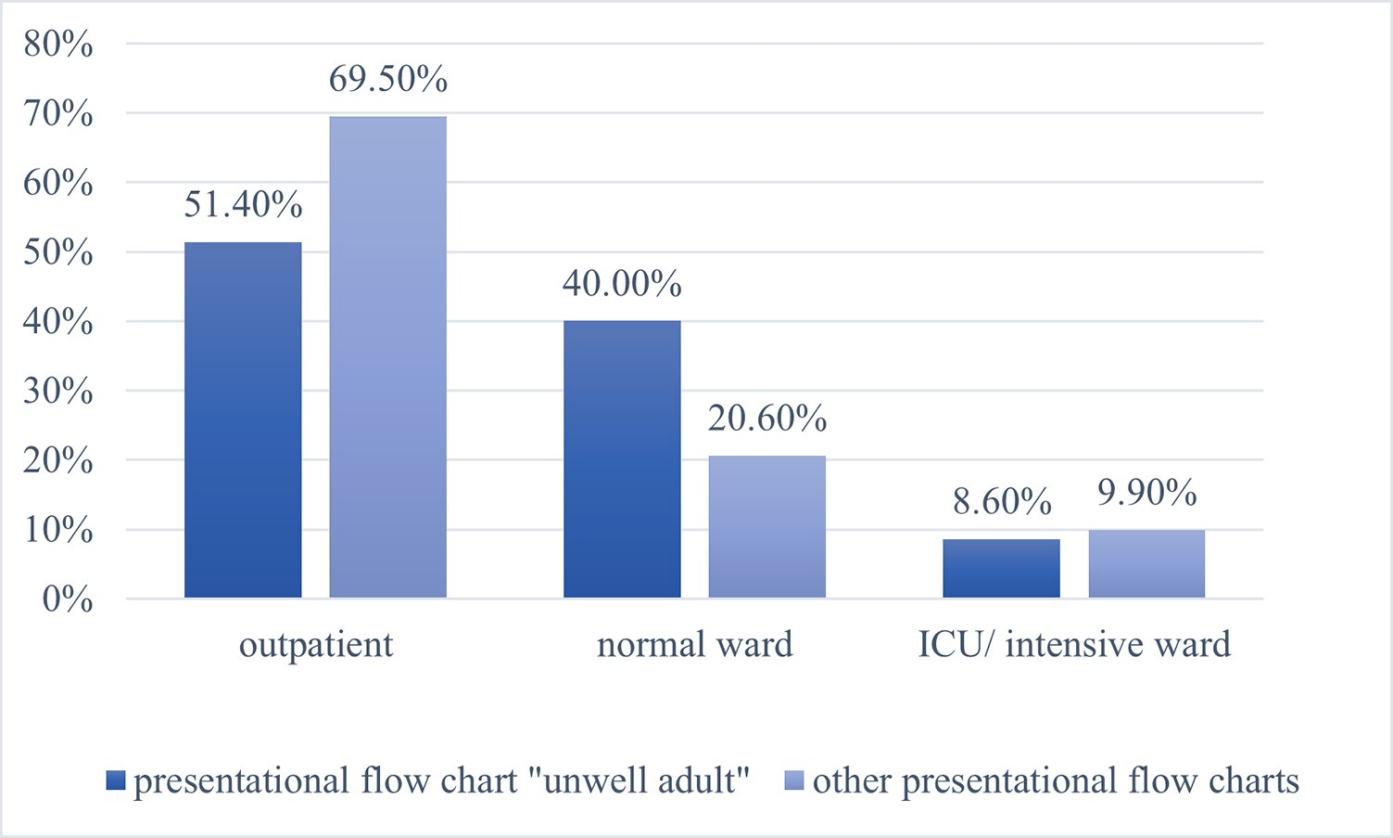
Level of care of the presentational flow chart “unwell adult” vs. the other presentational flow charts (p < 0.001).

In comparison with the other presentational flow charts, patients triaged with the “unwell adult” chart could be less frequently discharged as outpatients and had to be admitted to hospital. At that point, most “unwell adult” patients were transferred to a normal ward (40.0%) [Fig 4]. In patients triaged with the other presentational flow charts, this applied to only 20.6%. However, a transfer to an intermediate (IMC)/intensive care unit occurred less frequently in patients triaged according to the presentational flow chart "unwell adult" (8.6%) than in those triaged according to the other presentational flow charts (9.9%) (p < 0.001) [Fig 4].

**Fig 5:**
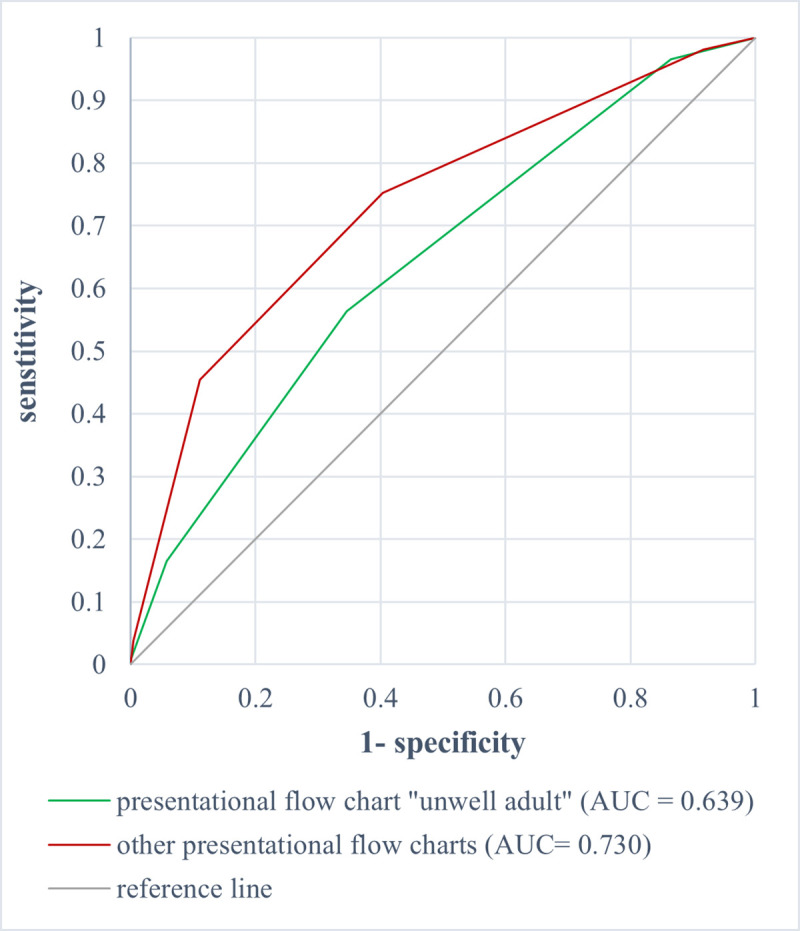
ROC analysis of MTS level and admission to hospital in general of patients aged ≥ 65 years for the presentational flow chart “unwell adult” (AUC = 0.639 [0.578–0.701]) vs. the other flow charts (AUC = 0.730 [0.714–0.746]).

Comparing the ROC analysis of the MTS levels in relation to hospital admission in general of older patients (aged ≥65 years), a significantly lower AUC of 0.639 (95% CI 0.578–0.701) was observed for the presentational flow chart "unwell adult" compared to the ROC analysis of the other presentational flow charts with 0.730 (95% CI 0.714–0.746) (p = 0,005) [Fig 5].

Comparison of the MTS level and admission to hospital of the younger patients (aged <65 years) for the presentational flow chart “unwell adult” with the other flow charts revealed no significant difference in the ROC analysis (p = 0.096).

**Fig 6:**
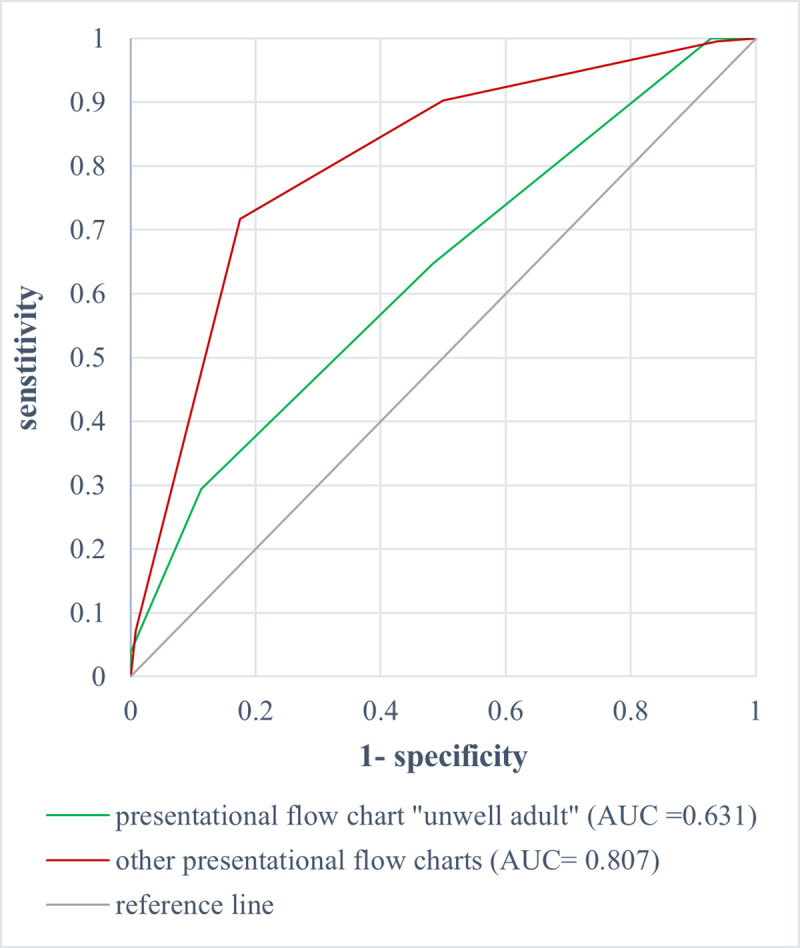
ROC analysis of MTS level and admission to ICU for patients aged ≥65 years for the presentational flow chart “unwell adult” (AUC = 0.631 [0.547–0.715] vs. the other flow charts (AUC = 0.807 [0.790–0.824]).

Furthermore, the predictive value of admission to ICU was lower in this unspecific presentational flow chart for patients aged ≥65 years than in the remaining flow charts and resulted in a lower AUC of 0.631 (95% CI 0.547–0.715) compared to an AUC of 0.807 (95% CI 0.790–0.824) (p< 0.001) [Fig 6].

Comparison of ROC analysis of the MTS in relation to admission to ICU of younger patients (aged <65 years) revealed a significantly lower AUC of 0.739 (95% CI 0.670–0.808) for the presentational flow chart “unwell adult” than ROC analysis of the other presentational flow charts with 0.839 (95% CI 0.819–0.859) (p = 0.007).

### In-hospital mortality

Regarding in-hospital mortality, we found a significant difference between the two comparison groups. While in-hospital mortality in the group with the remaining presentational flow charts was 1.5%, patients triaged with the presentational flow chart "unwell adult" had a mortality rate of 3.6% (p < 0.001). The most frequently applied ICD-10 diagnosis in the deceased patients was "neoplasms" (17.9%), followed by "certain infectious and parasitic diseases" and "diseases of the digestive system" (15.4% each). By far the most frequent diagnosis of patients who died in hospital and who were triaged according to the remaining presentational flow charts was "diseases of the circulatory system" with 55.0% (p < 0.001).

**Fig 7:**
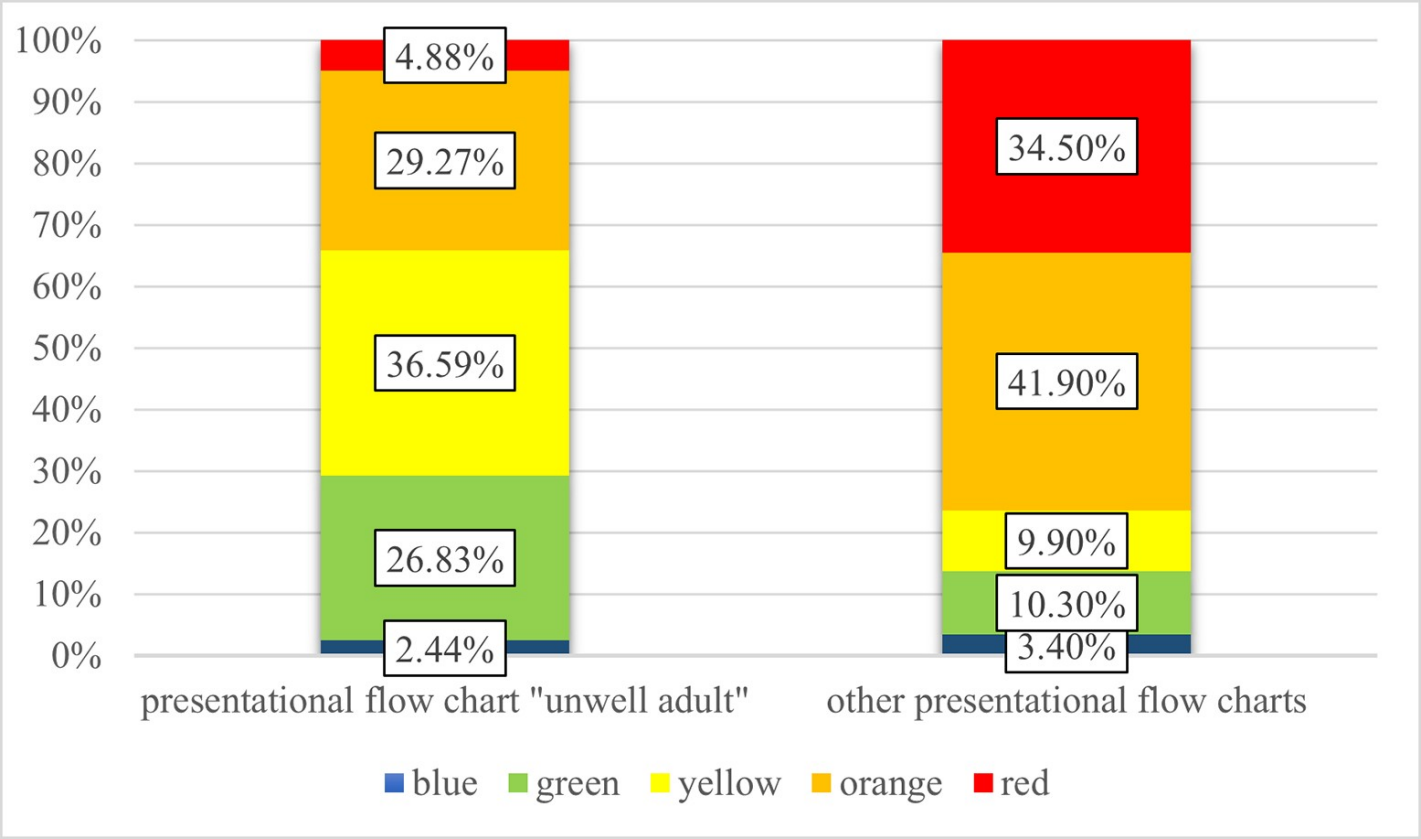
The different MTS urgency levels used in the in-hospital deceased patients triaged according to the presentational flow chart “unwell adult” vs. the deceased patients of the other presentational flow charts (p < 0.001).

Looking at the initial triage levels of the deceased patients, it was noticeable that in patients triaged with the "unwell adult" chart (n = 41), the higher triage levels "red" and "orange" were used much less frequently than in patients triaged with the other flow charts (34.15% for “unwell adults” vs. 76.4% for others). In contrast, the triage levels "yellow" and "green" were used much more frequently in patients triaged with the “unwell adult” chart, who died later during their hospital stay [Fig 7].

**Fig 8:**
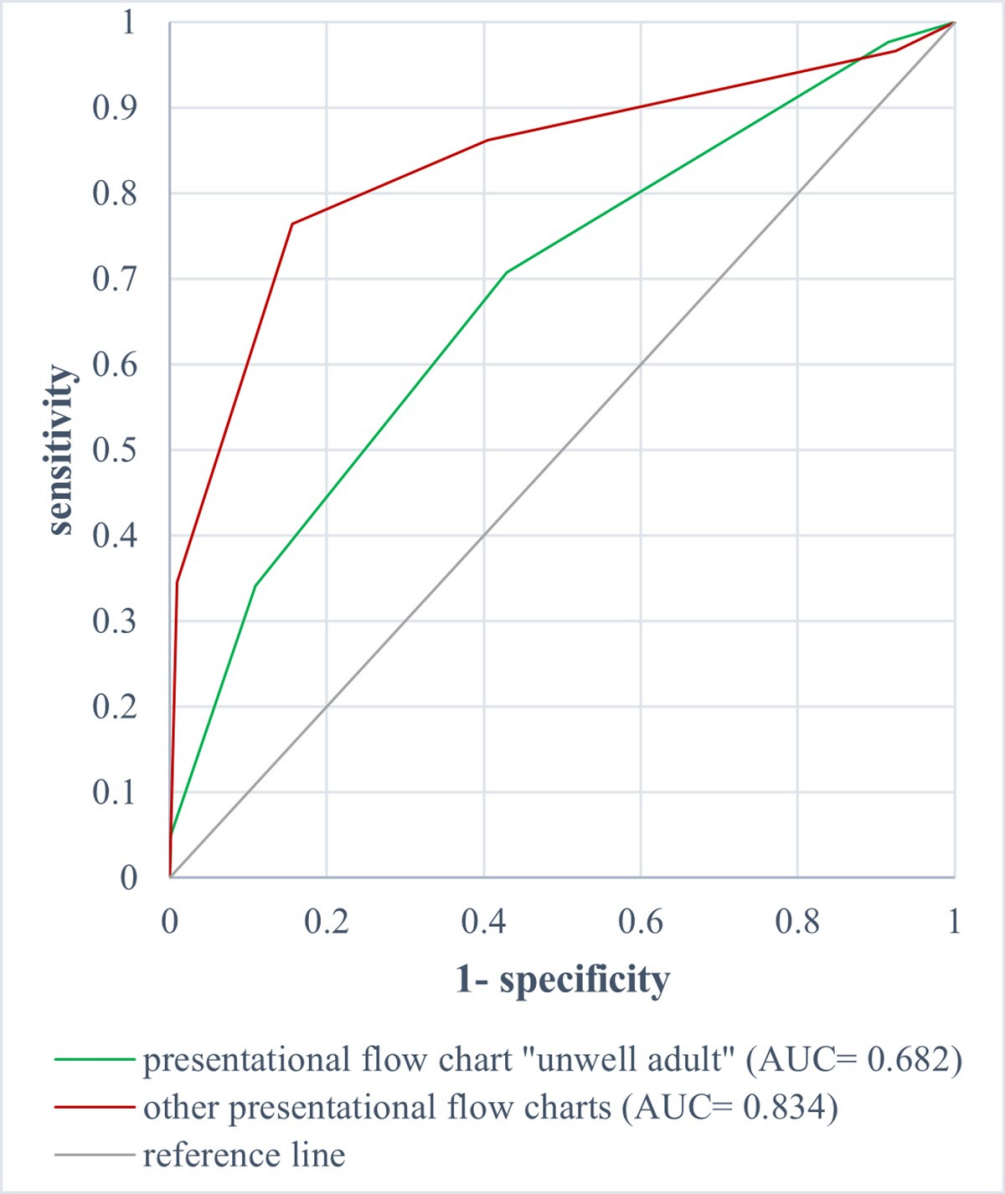
ROC analysis of the MTS level and in-hospital mortality for the presentational flow chart “unwell adult” (AUC = 0.682 [0.595–0.769]) vs. the other flow charts (AUC = 0.834 [0.799–0.869]).

ROC analysis of MTS triage level and mortality revealed an AUC of 0.682 (95% CI 0.595–0.769) for patients admitted to hospital and triaged with the “unwell adult” chart, vs. 0.834 (95% CI 0.799–0.869) for the other presentational flow charts (p = 0.001) [Fig 8].

## Discussion

The present study examines for the first time the initial assessment of a patient group triaged with the unspecific presentational flow chart "unwell adult" of the MTS in an ED of a tertiary hospital.

The results clearly show that the patient collective differs from the other emergency patients who were assessed with more specific presentational flow charts. In terms of construct validity, application of the MTS as a priority triage assessment tool shows that for the presentational flow chart "unwell adult", the MTS prediction is less accurate than for the other flow charts. Therefore, the present study contributes to health care research on triage in EDs, offering a starting point for adaption of structures and processes in the ED to improve secondary treatment paths and, ultimately, increase patient safety.

Triage scoring algorithms have been principally developed to support the triage nurse with objective criteria during the triage process. The selection of a presentational flow chart from the 52 available MTS flow charts requires a symptom-oriented nursing evaluation of the emergency patient. As shown, the flow chart "unwell adult" is the third most frequently used chart and thus very popular with MTS users. Analysis of ICD diagnoses reveals that the presentational flow chart "unwell adult" is preferably used for patients with nonspecific complaints. According to Nemec et al., nonspecific complaints are defined as the entity of complaints not part of the set of specific complaints for which evidence-based management protocols for emergency physicians (EPs) exist [10].

Considering the age of the patients in the “unwell adult” group, it is evident that the patients are significantly older than in the comparison group. Vanpee et al. came to the same conclusion in 2001, reporting that a large proportion of patients in the emergency room presents with unspecific symptoms. Particularly in older patients, often only diffuse and unspecific symptoms can be identified [18].

Several studies have shown that the MTS is a reliable and valid instrument for a first assessment of emergency patients in the ED [19–21]. A systematic review by Zachariasse et al. [21] shows a good validity of the MTS for hospitalization and admittance to ICU overall in different settings. However, the presentational flow chart "unwell adult" presents a risk of underestimating the severity of the diseases. Consequently, prediction of mortality or admission to the ICU was less accurate in the “unwell adult” group.

Interestingly, the AUC values of the other presentational flow charts from a previous investigation were confirmed in this study [1]. Our data are also congruent with the results of a recent systematic review by Kemp et al. [22], who, with an odds ratio of 2.2, were able to show that patients with unspecific complaints were classified as significantly less urgent than patients with specific symptom. According to the authors, an explanation for the research results can be found in the indicator levels of the presentational flow charts and thus in the symptoms presented by the patients upon arrival at the ED. The more pronounced the patients’ symptoms are upon arrival at the hospital, the higher the urgency level in the MTS.

With increasing age, specific symptoms of even serious illnesses become rarer and instead manifest as a worsened general condition (generalized weakness) [23]. Studies have shown that over 50% of elderly patients in the emergency room with nonspecific symptoms suffered from an acute illness [24,25]. The CHARITEM study demonstrated that one out of 20 emergency patients with an unclear leading symptom dies [11]. If necessary, age must be considered as an independent risk factor in triage. Ginsburg et al. reported that older emergency patients with the same chief complaint and triage classification had significantly higher ED resource utilization, hospitalization rates, and mortality compared with younger patients [26]. Consideration of a patient’s age could further increase the validity of the presentational flow chart "unwell adult". Thus, a new discriminator "old or vulnerable age" could be added to the indicator list of the presentational flow chart "unwell adult". Further studies are needed to clarify the extent to which the performance of this MTS flow chart improves when age is added as an independent risk factor.

Why, even in the case of serious illnesses, nonspecific symptoms occur significantly more often in old age than during younger phases of life may be explained two-fold. Firstly, the performance of all physical systems decreases with increasing age. This could also affect the intrathoracic and intraabdominal perception of pain and thus explain why older patients feel little or no pain even in the case of a heart attack or severe intraabdominal inflammation. Secondly, the decrease in performance in elderly patients also affects the defense mechanisms [27]. These facts go along with the ICD-10 chapters we found represented in the group of deceased patients.

If these findings are applied to the discriminators of the MTS presentational flow chart "unwell adult", the following points become apparent. Principally, there are already discriminators in the individual triage levels that target nonspecific complaints. It is possible that their level of urgency is not high or comprehensive enough. Discriminators that ask for more unspecific symptoms, such as chills, lack of appetite, mottled skin, "looking ill" and diarrhea analogues in the Emergency Severity Index (ESI), could be helpful here. Based on scores, such as the qSOFA score in the MTS, we suggest including a so-called NSC (nonspecific complaint) score to the indicators in order to improve the representation of nonspecific complaints in the presentational flow chart "unwell adult". The above-mentioned unspecific symptoms should be included individually in the NSC score to allow their full consideration. In addition, functional decline, psychosocial dysfunction and the impact of comorbidities must be taken into account [28,29], as does evaluation with regard to adverse drug reaction [30].

In this context, electronic emergency triage systems (e-triage) may be able to improve the quality of triage. Electronic triage systems use simple standardized patient information routinely collected at triage to predict risk for critical outcome. Based on the patient’s individual estimated risk, the level of urgency is determined. Dugas et al. published a multicentric study of patient data from more than 25,000 emergency visits, which demonstrated that emergency patients with higher severity of illness were more reliably identified using e-triage [31].

Finally, the awareness for the group of patients triaged with the presentational flow chart "unwell adult" must improve. Every member of staff in the ED must be aware that deterioration of the general condition is not a sign of aging but can be an expression of illness. Beglinger et al. were able to prove that in patients with unspecific symptoms, the first clinical impression is related to morbidity and mortality [32]. In this context, two points seem important. The first one is compliance with the maximum time limit for first contact with a doctor. Our data show that there is still room for improvement, especially in the higher priority levels. Better training of emergency nurses and physicians in recognizing atypical clinical presentation and identification of patients at high risk could improve this process [24]. The second point applies to the level of care. The significantly lower values of the AUC in our ROC analysis in relation to the level of care for the “unwell adult” flow chart show the lower prediction for this unspecific presentational flow chart.

This is also noticeable considering the significant difference in waiting times for both comparison groups at the triage level "orange". While the level of awareness of patients with, e.g., chest pain/ ACS is significantly higher due to guidelines, which stipulate exact time specifications, patients triaged with the presentational flow chart "unwell adult" are perceived as to be in less threatening circumstances and are thus examined later.

Misch et al. came to the same conclusions in their observational study [33], showing that patients with NSC are at high risk for inappropriate disposition planning. This may add to the increased risk for adverse health outcomes. The accuracy regarding hospitalization of patients with acute and serious conditions increases after observation, suggesting a potential benefit of an observation unit [33]. Therefore, especially in the case of a rapidly developed deterioration of the general condition in older patients, one should be alerted and generously set the indication for admission to an adequate level of care. In this respect, deterioration of the general condition is principally treatable.

## Conclusion

In conclusion, the presentational flow chart "unwell adult" is frequently used by triage nurses for the initial assessment of patients. Patient characteristics assessed with this presentational flow chart differ significantly from those assessed with the more specific presentational flow charts of the MTS. Presentation in the emergency room with so-called nonspecific complaints is reflected in the patient characteristics, symptoms, and diagnosis. In light of demographic developments that have led to an aging population, the presentational flow chart “unwell adult” will play an increasingly important role in the future.

The outcome data found by studies of patients with unspecific complaints speak for themselves. The user of the MTS is fortunate to have a presentational flow chart that defines itself by the unspecific presentation of the emergency patient. However, this "blessing" is accompanied by a small "curse". The quality of the initial assessment in terms of a well-functioning triage priority assessment tool is less accurate than the performance of the MTS described in the literature. Necessary modifications to the indicator level of the presentational flow chart "unwell adult" must be investigated in further prospective studies.

## Limitation

Our study has several limitations. Since this is a monocentric, retrospective study, the generalization of the findings may be limited. A certain selection bias in the patient population cannot be ruled out—due to special university subjects, such as ophthalmology, ENT, or dermatology.

Moreover, we were not able to list comorbidities of each patient and thus, we cannot rule out that comorbidities could have influenced the outcome of the patients in our study.

Ultimately, it must be taken into consideration that our study did not include the course of diseases after discharge from hospital.
